# Supplementary material for: Possibility for Prevention of Type 2 Diabetes Mellitus and Dementia Using Three Kinds of Brown Rice Blends after High-Pressure Treatment
Source: Foods. 2022 Mar 12;11(6):818. doi: 10.3390/foods11060818 (PMC8947517; doi:10.3390/foods11060818)
Supplement: Supplementary file 1 [file foods-11-00818-s001.zip › foods-1610998-supplementary.pdf]

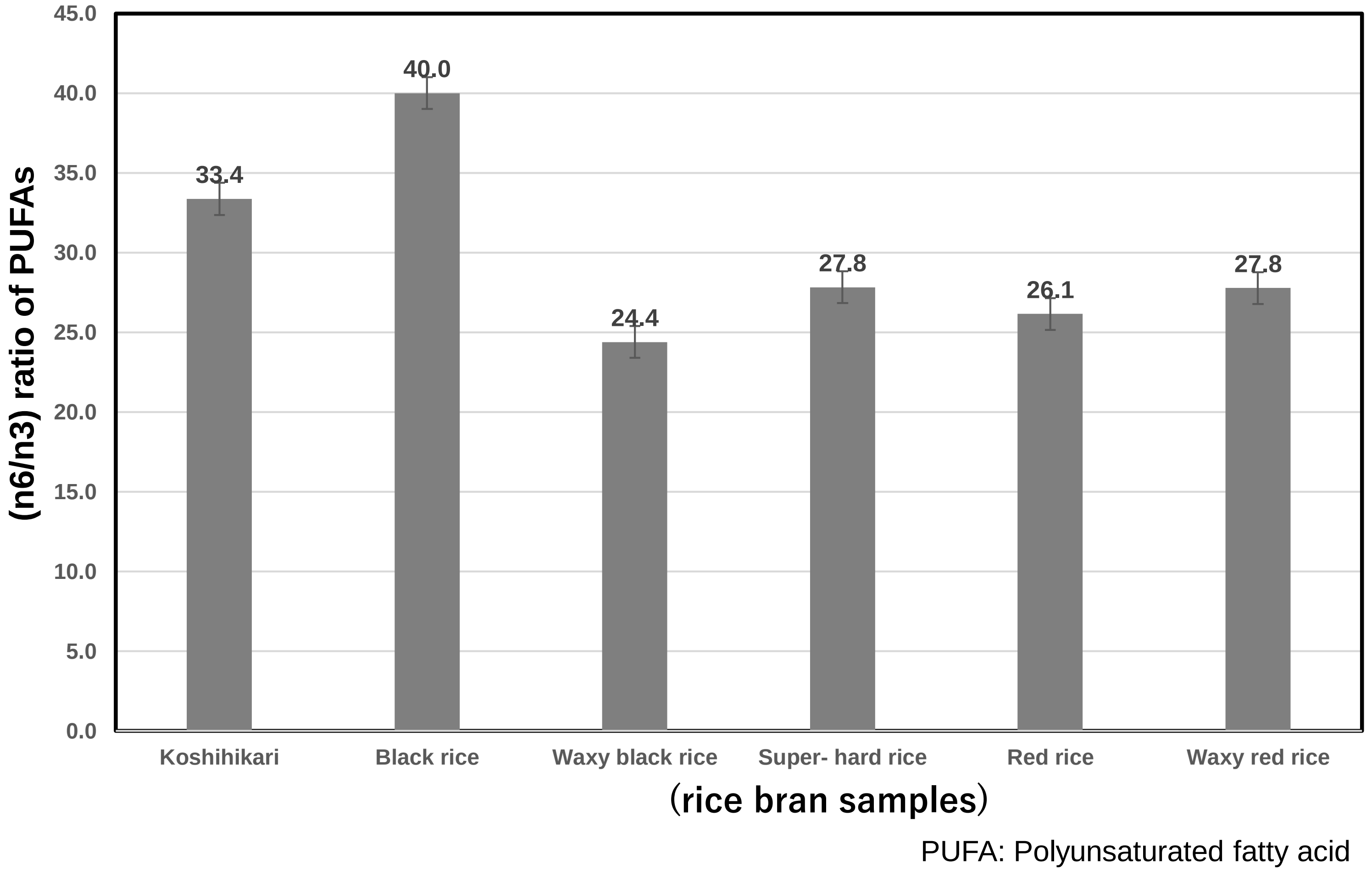

Supplemental Fig S1. n6/n3 ratio of PUFAs of 6 kinds of japonica rice bran

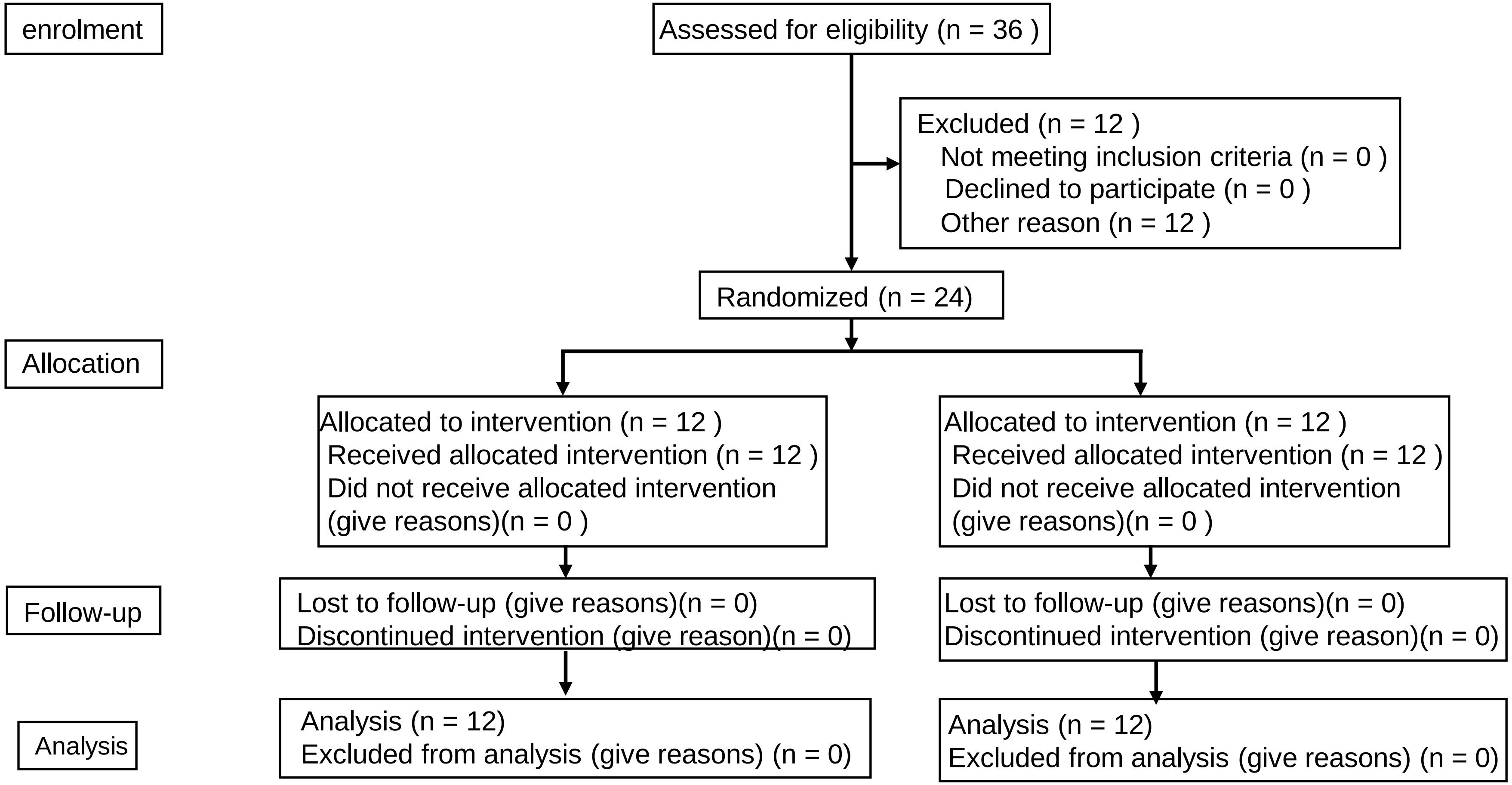

Supplemental Fig S2. Flow diagram of the progress through the phases of a parallel randomized trial of two groups.

Supplemental Table S1. The principal ingredients of 2 kinds of cooked rice for Human test.

	Test meal	Control meal
Daily intake (g)	190.0	200.0
Carbohydrates (g)	64.0	66.8
Protein (g)	6.3	4.8
Lipid (g)	2.9	1.0
Moisture (g)	116.0	127.0
Mineral (mg)	1.1	0.2
Dietary fiber (g)	16.2	0.8
Anthocyanin (mg)	15.2	0.0
Energy (Kcal)	268.0	294.0

Supplemental Table S2. Difference in appearance among of cooked rice of ordinary white rice, brown rice and wax-free brown rice.

	WB<br>Whiteness	L*<br>Brightness	a*<br>Redness	b*<br>Yellowish
**Cooked rice of white rice of Koshihikari**	**25.3 ± 1.0 a**	**58.2 ± 1.5 a**	**-3.2 ± 0.0 a**	**1.4 ± 0.9 a**
**Cooked rice of brown rice of Koshihikari**	**16.4 ± 0.1 b**	**54.4 ± 0.1 b**	**-1.6 ± 0.2 b**	**11.9 ± 0.1 b**
**Cooked rice of wax-free brown rice of Koshihikari**	**22.5 ± 4.3 a**	**59.7 ± 2.7 a**	**-2.1 ± 0.7 ab**	**9.1 ± 0.5 c**

Within each measure (WB, L*, a*, and b*) in the same column, different letters (a, b, etc) denote statistically significant differences.
